# A multicenter explainable machine learning analysis of autoimmune disease comorbidity in ankylosing spondylitis

**DOI:** 10.3389/fimmu.2026.1775877

**Published:** 2026-02-26

**Authors:** Jichong Zhu, Chengqian Huang, Yang Lin, Tianyou Chen, Lei Ren, Jiarui Chen, Jiang Xue, Hao Li, Hong Cheng, Xinli Zhan, Chong Liu

**Affiliations:** 1 The First Affiliated Hospital of Guangxi Medical University, Nanning, China; 2 Affiliated Hospital of Youjiang Medical University for Nationalities, Spinal Orthopedic Surgery, Baise, China; 3 Key Laboratory of Molecular Pathology in Tumors of GuangxiHigher Education Institutions, Baise, China; 4 Guilin People’s Hospital, Guilin, China; 5 The Second Affiliated Hospital of Guangxi Medical University, Nanning, China; 6 Nanxishan Hospital of Guangxi Zhuang Autonomous Region (The Second People’s Hospital of Guangxi Zhuang Autonomous Region), Department of Trauma Orthopaedics and Hand Surgery, Guilin, China

**Keywords:** ankylosing spondylitis, autoimmune comorbidity, LightGBM, machine learning, SHAP

## Abstract

**Background:**

Ankylosing spondylitis (AS) frequently coexists with other autoimmune diseases, leading to increased clinical heterogeneity and diagnostic complexity. Early identification of autoimmune comorbidity in AS remains challenging in routine practice.

**Methods:**

A multicenter, retrospective, cross-sectional study was conducted, where clinical and laboratory data were collected from three independent tertiary centers between 2012 and 2025. Patients were classified into three groups: AS alone, autoimmune diseases alone, and AS with autoimmune comorbidities. Routinely available variables, including demographic characteristics, systemic inflammatory indices, hematological parameters, and liver and renal function markers, were analyzed. Multiple machine learning algorithms were developed for two clinically relevant classification tasks: AS alone vs. AS with autoimmune comorbidities, and autoimmune diseases alone vs. AS with autoimmune comorbidities. Model performance was evaluated using AUC, calibration, decision curve analysis, and clinical impact curves. SHapley Additive exPlanations (SHAP) were applied to enhance interpretability.

**Results:**

Among all models, LightGBM consistently demonstrated superior and stable performance across discrimination, calibration, and clinical utility metrics. In distinguishing AS alone from AS with autoimmune comorbidities, key contributors included age, gender, renal function–related markers (eGFR, CysC, BUN, UA), and protein and hepatobiliary indices (ALB, DBIL). In comparisons between autoimmune diseases alone and AS with autoimmune comorbidities, SHAP highlighted metabolic- and synthesis-related features (GLOB, PREALB, CHE, ALP), acid–base balance (HCO_3_), and inflammatory activity (ESR). These patterns suggest that AS-associated autoimmune comorbidity represents a distinct systemic inflammatory–metabolic phenotype rather than a simple amplification of inflammation.

**Conclusions:**

Using routinely available clinical data, an explainable machine learning framework enables accurate identification and characterization of autoimmune comorbidity in AS. This approach has practical potential for early risk stratification and clinical decision support in real-world settings.

## 1 Introduction

Ankylosing spondylitis (AS) is a chronic inflammatory disorder primarily affecting the sacroiliac joints and spine, with a strong association to the HLA-B27 gene (1). It predominantly affects young males, and its prevalence varies by ethnicity and geography. In addition to axial involvement, AS is commonly accompanied by extra-articular manifestations, including uveitis, inflammatory bowel disease(IBD), psoriasis, cardiovascular complications, and osteoporosis (2, 3). Notably, AS patients may also develop comorbid autoimmune diseases such as psoriatic arthritis and IBD, indicating a pattern of systemic immune dysregulation (4). However, the underlying mechanisms linking AS to these coexisting autoimmune conditions remain insufficiently explored and require further investigation.

Patients with AS may present with concomitant autoimmune diseases, which differ in affected organs and clinical manifestations. For example, rheumatoid arthritis (RA) primarily involves peripheral small joints; systemic lupus erythematosus (SLE) is characterized by multisystem involvement and autoantibody production; Sjögren’s syndrome mainly affects exocrine glands; and IBD is predominantly associated with chronic inflammation of the gastrointestinal tract (5). Despite these phenotypic differences, these conditions are all immune-mediated chronic inflammatory disorders, sharing common features such as immune dysregulation, aberrant cytokine networks, and genetic susceptibility (6). The presence of immune comorbidities can substantially increase disease complexity and systemic inflammatory burden in AS patients, leading to higher disease activity, more frequent extra-articular manifestations, and reduced therapeutic responses, thereby adversely affecting long-term prognosis and quality of life (7). Therefore, early identification of immune comorbidities and exploration of relevant biomarkers or clinical indicators are crucial for achieving precise disease assessment and individualized treatment strategies in AS.

Machine learning has demonstrated substantial advantages in the diagnosis and research of immune-mediated diseases, particularly in handling high-dimensional and heterogeneous clinical and biological data, where it can capture complex nonlinear relationships to enable earlier and more accurate diagnosis and prognostic assessment (8). Moreover, machine learning facilitates the efficient integration of large-scale, multi-source data, including genomics, imaging, and electronic health records, uncovering associations that are often inaccessible to conventional analytical approaches (9). However, the limited interpretability of complex models has raised concerns regarding their clinical applicability. In this context, explainable techniques such as SHapley Additive exPlanations (SHAP) quantify the contribution of individual features to model predictions, enhancing transparency and reliability and thereby supporting the trustworthy implementation of machine learning in clinical practice (10).

This study aimed to compare clinical characteristics among three populations—patients with AS alone, patients with other autoimmune diseases without AS, and patients with AS comorbid with other autoimmune diseases—in order to identify early indicators of AS-related immune comorbidity. Clinical data were collected from three independent centers and comprised multidimensional variables, including routine blood parameters, erythrocyte sedimentation rate (ESR), liver and renal function indices, and systemic inflammatory markers. Multiple machine learning algorithms were applied for feature selection and classification model development, with particular emphasis on discriminative patterns between “AS alone versus AS with comorbid autoimmune diseases” and “other autoimmune diseases versus AS with comorbid autoimmune diseases.”

Furthermore, SHAP were employed to enhance model interpretability by quantifying the contribution of individual features to classification outcomes, thereby elucidating key variables associated with comorbidity and identifying potential biomarkers and clinical features for the early detection and stratified management of autoimmune comorbidities in AS.

## 2 Materials and methods

### 2.1 Study design and population

A multicenter, retrospective, cross-sectional study was conducted, utilizing clinical data collected from three independent tertiary medical centers. Patients were consecutively enrolled between December 2012 and January 2025. All participants underwent systematic evaluation by rheumatologists, and definitive diagnoses were established based on integrated assessments of clinical manifestations, laboratory findings, and imaging examinations, in accordance with internationally recognized or widely accepted diagnostic criteria.

According to disease status, patients were classified into three groups. The first group comprised patients with AS alone. The diagnosis of AS was based on the ASAS (Assessment of SpondyloArthritis International Society) classification criteria and was independently confirmed by at least two experienced rheumatologists. Diagnostic evaluation incorporated typical clinical features, such as chronic inflammatory back pain and limitation of spinal or sacroiliac joint mobility, in line with the ASAS clinical criteria. Imaging evidence of sacroiliitis or structural changes on radiography, computed tomography (CT), or magnetic resonance imaging (MRI), consistent with the ASAS imaging criteria, were also considered. Additionally, relevant laboratory findings, including HLA-B27 testing, were evaluated as part of the diagnostic process. Patients in this group had no concomitant autoimmune diseases (11).

The second group included patients with autoimmune diseases other than AS, without clinical or imaging evidence of AS. These autoimmune conditions included psoriasis, inflammatory bowel disease, uveitis, dermatitis, rheumatoid arthritis, systemic lupus erythematosus, Sjögren’s syndrome, Crohn’s disease, amyopathic dermatomyositis, vasculitis, Behçet’s disease, systemic sclerosis, and other immune-mediated disorders. All diagnoses were made according to the corresponding internationally recognized or nationally accepted diagnostic criteria, including the CASPAR criteria for psoriatic arthritis, the 2010 ACR/EULAR criteria for rheumatoid arthritis, the 2019 EULAR/ACR criteria for systemic lupus erythematosus, the 2016 ACR/EULAR criteria for Sjögren’s syndrome, the 2012 Chapel Hill criteria for vasculitis, and the 1990 International Criteria for Behçet’s disease, among others. These criteria integrate clinical features, laboratory tests, imaging studies, and specific antibody markers to ensure accurate diagnosis and consistency.

The third group consisted of patients with AS comorbid with other autoimmune diseases. These patients fulfilled the diagnostic criteria for AS as well as for at least one additional autoimmune disease, with each diagnosis independently confirmed by specialist physicians.

### 2.2 Clinical and laboratory features

Clinical and laboratory data were collected at the time of initial evaluation or hospital admission. Demographic variables included age and gender. Systemic inflammatory indices comprised the systemic immune-inflammation index (SII), systemic inflammation response index (SIRI), neutrophil-to-lymphocyte ratio (NLR), platelet-to-lymphocyte ratio (PLR), platelet-to-neutrophil ratio (PNR), and neutrophil-to-monocyte ratio (NMR).

Routine hematological parameters included white blood cell count (WBC), red blood cell count (RBC), hemoglobin (Hb), hematocrit (HCT), mean corpuscular volume (MCV), mean corpuscular hemoglobin (MCH), mean corpuscular hemoglobin concentration (MCHC), platelet count (PLT), mean platelet volume (MPV), platelet distribution width (PDW), and plateletcrit (PCT). Differential leukocyte counts included neutrophils (NE), lymphocytes (LY), monocytes (MO), eosinophils (EO), and basophils (BA).

Inflammatory activity was assessed using the erythrocyte sedimentation rate (ESR).

Liver function parameters included total bilirubin (TBIL), direct bilirubin (DBIL), indirect bilirubin (IBIL), the DBIL/TBIL ratio, total protein (TP), albumin (ALB), globulin (GLOB), the albumin-to-globulin ratio (ALB/GLOB), gamma-glutamyl transferase (GGT), total bile acid (TBA), aspartate aminotransferase (AST), alanine aminotransferase (ALT), the AST/ALT ratio, alkaline phosphatase (ALP), prealbumin (PREALB), and cholinesterase (CHE).

Renal function and related metabolic parameters included blood urea nitrogen (BUN), creatinine (Cr), uric acid (UA), bicarbonate (HCO_3_), estimated glomerular filtration rate (eGFR), and cystatin C (CysC).

### 2.3 Data preprocessing

Prior to model development, clinical data were preprocessed to ensure data quality. Patient records with missing values were excluded, and only patients with complete clinical and laboratory data were included in the analysis. The data were then organized and analyzed separately according to their originating centers.

Continuous variables were uniformly processed before analysis to minimize the influence of different measurement scales on model training, while categorical variables were encoded into numerical formats suitable for machine learning algorithms. The same preprocessing procedures were applied across all centers to ensure comparability and robustness of the analytical results (12, 13).

After data screening, the final study population comprised three disease groups across the three centers. In the AS-alone group, 326 patients were included from Center 1, 108 from Center 2, and 110 from Center 3. In the group with autoimmune diseases other than AS, 603 patients were enrolled from Center 1, 201 from Center 2, and 201 from Center 3. In the AS with autoimmune comorbidity group, 343 patients were included from Center 1, 114 from Center 2, and 115 from Center 3.

### 2.4 Machine learning models

After data preprocessing, multiple machine learning–based binary classification models were developed using clinical and laboratory variables to discriminate between different disease states. The algorithms applied included Naïve Bayes, discriminant analysis, logistic regression, LASSO, partial least squares, k-nearest neighbors, support vector machines, artificial neural networks, random forest, gradient boosting and other boosting methods, as well as ensemble learning models such as LightGBM and CatBoost.

Model training was primarily performed on the training dataset with repeated five-fold cross-validation to assess model stability. Model performance was evaluated in both the training and testing datasets using the area under the receiver operating characteristic curve (AUC), sensitivity, specificity, accuracy, F1 score, and Youden index. Receiver operating characteristic (ROC) curves and decision curve analysis (DCA) were used to compare discriminative ability and potential clinical utility across models (14, 15). The model with the highest AUC in the testing dataset was selected for subsequent analyses.

### 2.5 SHAP analysis

To enhance model interpretability, SHAP were applied to the optimal machine learning model selected based on test-set performance. SHAP was used to quantify the contribution of individual clinical and laboratory features to model predictions at both global and individual levels, thereby elucidating the model’s decision-making process (8).

For tree-based models (e.g., LightGBM and CatBoost), SHAP values were computed using model-specific built-in methods, while a model-agnostic kernel-based SHAP approach was used for the remaining models. Feature importance was assessed using mean absolute SHAP values, and SHAP summary and dependence plots were generated to visualize the direction and magnitude of feature effects (16). In addition, waterfall or force plots were used to provide intuitive explanations for representative individual predictions.

### 2.6 Feature engineering and feature selection process

In this study, we performed feature engineering and feature selection through the following steps: (1) Data Preprocessing: Missing values were handled, and continuous variables were standardized to mitigate the effects of different scales. Categorical variables (e.g., gender) were converted into numerical format. (2) Feature Selection Method: A total of 48 variables were initially considered. Machine learning algorithms were applied to assess the importance of features in clinical and laboratory data. The most important features for distinguishing AS from autoimmune comorbidities were selected. (3) SHAP Analysis: To enhance model interpretability, SHAP (Shapley Additive Explanations) was used to quantify the contribution of each feature to the model’s predictions. This analysis helped confirm the clinical and biological relevance of the selected features. (4) Feature Selection Criteria: The selected features demonstrated strong statistical correlation and were aligned with clinical relevance. Feature selection ensured that the model was both effective in prediction and resistant to overfitting.

## 3 Results

### 3.1 Baseline characteristics and laboratory differences among the three groups

**Figure 1:**
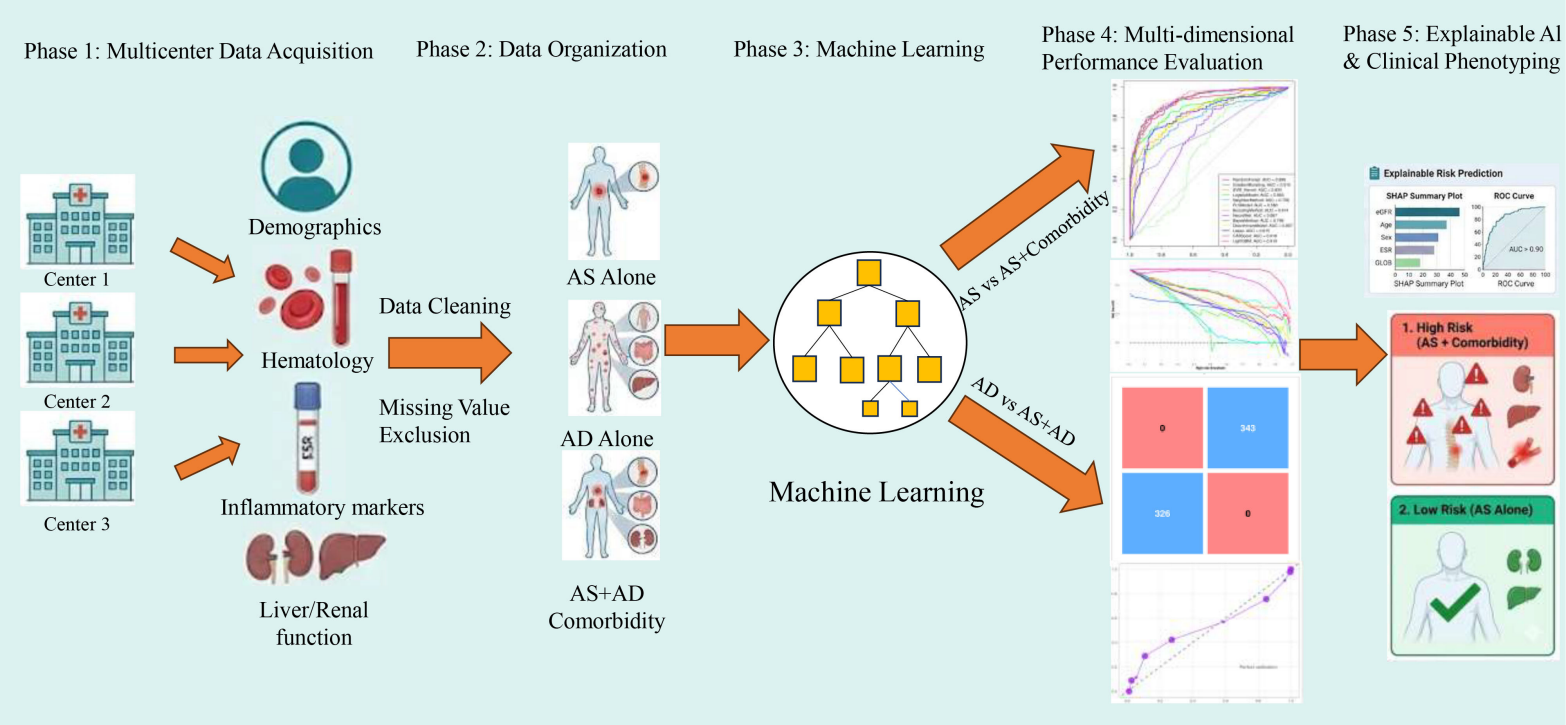
Complete research flowchart.

After merging data from the three centers, a total of 2,121 patients were included in the analysis, comprising 544 patients with AS alone, 1,005 patients with autoimmune diseases without AS, and 572 patients with AS combined with other autoimmune diseases. Comparisons of demographic characteristics, systemic inflammatory indices, hematological parameters, liver function, and renal function–related variables among the three groups are summarized in **Tables 1**, **2**, **3**, **4**. The complete research flowchart is shown in **Figure 1**.

**Table 1:** Demographic characteristics and systemic inflammatory indices among patients with ankylosing spondylitis alone, autoimmune diseases alone, and ankylosing spondylitis with autoimmune comorbidities.

Type	AS (N = 544)	Immunological diseases (N = 1005)	Immunological diseases+AS (N = 572)	p-value
Male	458 (84.2%)	385 (38.3%)	253 (44.2%)	<0.01
Female	86 (15.8%)	620 (61.7%)	319 (55.8%)	
Age	35.00 (29.00, 41.00)	52.00 (36.00, 61.00)	52.00 (36.00, 63.00)	<0.01
ESR,mm/h	31.50 (17.00, 54.25)	17.00 (8.00, 34.00)	28.00 (14.00, 49.00)	<0.01
SII	803.30 (495.82, 1195.93)	545.97 (373.07, 920.00)	745.11 (494.11, 1252.68)	<0.01
SIRI	1.51 (1.00, 2.37)	1.11 (0.72, 1.97)	1.42 (0.92, 2.25)	<0.01
NLR	2.52 (1.84, 3.45)	2.16 (1.53, 3.14)	2.42 (1.74, 3.36)	<0.01
PLR	151.64 (118.30, 203.17)	134.13 (102.66, 184.50)	150.08 (113.88, 205.17)	<0.01
PNR	60.99 (49.07, 78.41)	62.65 (47.63, 83.93)	64.26 (50.35, 80.88)	0.20
NMR	8.18 (6.53, 10.43)	7.66 (6.22, 9.95)	8.11 (6.45, 10.25)	<0.01

**Table 2:** Comparison of routine hematological parameters among the three study groups.

Type	AS (N = 544)	Immunological diseases (N = 1005)	Immunological diseases+AS (N = 572)	p-value
WBC, ×10^9/L	8.05 (6.90, 9.53)	7.20 (5.84, 8.73)	7.88 (6.71, 9.38)	<0.01
RBC, ×10^12/L	4.81 (4.42, 5.25)	4.57 (4.13, 5.06)	4.92 (4.45, 5.29)	<0.01
Hb, g/L	131.30 (119.00, 142.93)	129.00 (115.80, 142.20)	132.10 (117.95, 144.00)	0.07
HCT, %	0.40 (0.37, 0.43)	0.39 (0.36, 0.43)	0.41 (0.36, 0.44)	<0.01
MCV, fL	84.17 (78.17, 89.02)	87.60 (82.36, 91.00)	84.60 (78.04, 88.27)	<0.01
MCH, pg	27.84 (25.55, 29.53)	29.02 (27.06, 30.40)	27.79 (24.94, 29.47)	<0.01
MCHC, g/L	328.00 (319.75, 334.70)	329.00 (322.00, 336.20)	326.60 (317.45, 334.00)	<0.01
PLT, ×10^9/L	308.35 (254.75, 379.15)	266.00 (216.50, 330.00)	312.25 (253.85, 389.18)	<0.01
MPV, fL	7.95 (7.36, 8.76)	8.56 (7.76, 9.50)	8.25 (7.42, 9.20)	<0.01
PDW, %	0.16 (0.16, 0.17)	0.16 (0.12, 0.17)	0.16 (0.11, 0.17)	<0.01
NE#	5.10 (4.01, 6.50)	4.12 (3.19, 5.60)	4.97 (3.90, 6.21)	<0.01
LY#	2.00 (1.69, 2.40)	1.97 (1.51, 2.50)	2.01 (1.64, 2.54)	0.07
MO#	0.60 (0.49, 0.78)	0.54 (0.42, 0.69)	0.60 (0.48, 0.76)	<0.01
EO#	0.12 (0.08, 0.22)	0.14 (0.08, 0.25)	0.12 (0.07, 0.23)	0.03
BA#	0.03 (0.02, 0.05)	0.03 (0.02, 0.05)	0.04 (0.02, 0.05)	0.04
RDW-CV, %	0.14 (0.13, 0.15)	0.14 (0.13, 0.15)	0.14 (0.13, 0.15)	<0.01
PCT	0.25 (0.21, 0.30)	0.23 (0.19, 0.28)	0.26 (0.21, 0.31)	<0.01

**Table 3:** Comparison of liver function– and protein metabolism–related parameters among the three study groups.

Type	AS (N = 544)	Immunological diseases (N = 1005)	Immunological diseases+AS (N = 572)	p-value
TBIL, μmol/L	7.80 (6.00, 10.60)	8.80 (6.13, 13.00)	11.00 (7.40, 11.00)	<0.01
DBI, μmol/L	2.60 (1.80, 3.40)	2.88 (2.10, 4.00)	4.16 (2.50, 4.16)	<0.01
TBIL, μmol/L	5.40 (3.80, 7.12)	6.00 (3.93, 9.04)	6.84 (4.88, 6.84)	<0.01
DBIL/TBIL	0.30 (0.29, 0.40)	0.30 (0.28, 0.40)	0.35 (0.30, 0.35)	<0.01
TP, g/L	75.80 (71.20, 79.60)	71.00 (65.40, 76.00)	74.60 (74.07, 76.43)	<0.01
ALB, g/L	42.00 (38.68, 44.90)	41.20 (37.00, 44.60)	41.10 (40.70, 42.90)	<0.01
GLB, g/L	33.40 (29.00, 37.82)	29.20 (25.80, 33.50)	33.40 (31.90, 34.00)	<0.01
ALB/GLB	1.30 (1.00, 1.50)	1.40 (1.20, 1.60)	1.30 (1.20, 1.30)	<0.01
GGT, U/L	24.00 (18.00, 40.00)	23.00 (16.00, 37.00)	39.10 (22.00, 39.10)	<0.01
TBA, μmol/L	3.40 (2.10, 5.90)	4.20 (2.44, 7.10)	7.19 (3.10, 7.19)	<0.01
AST, U/L	20.00 (16.00, 25.00)	22.00 (18.00, 29.00)	26.50 (20.00, 26.50)	<0.01
ALT, U/L	18.00 (12.75, 28.00)	18.00 (12.00, 27.00)	25.00 (17.00, 25.00)	<0.01
AST/ALT ratio	1.10 (0.80, 1.50)	1.20 (0.90, 1.70)	1.30 (1.00, 1.30)	<0.01
ALP, U/L	98.00 (75.00, 125.00)	78.00 (61.00, 101.00)	103.30 (88.00, 103.30)	<0.01
PREALB,mg/L	213.95 (175.90, 260.20)	242.50 (194.40, 293.10)	228.30 (209.65, 244.22)	<0.01
CHE, U/L	8199.00 (7240.00, 9523.00)	8121.00 (6893.00, 9402.00)	8383.60 (8134.75, 8605.00)	0.02

**Table 4:** Comparison of renal function and metabolic parameters among the three study groups.

Type	AS (N = 544)	Immunological diseases (N = 1005)	Immunological diseases+AS (N = 572)	p-value
BUN, mmol/L	4.33 (3.56, 5.35)	4.52 (3.68, 5.80)	4.76 (4.27, 4.79)	<0.01
Cr, μmol/L	69.00 (58.00, 79.00)	66.00 (54.00, 81.00)	75.20 (66.75, 75.20)	<0.01
UA, μmol/L	332.00 (262.75, 394.25)	322.00 (254.00, 399.00)	342.40 (325.00, 356.00)	<0.01
HCO3-, mmol/L	26.11 (24.40, 27.80)	24.99 (23.00, 26.80)	25.80 (25.30, 25.80)	<0.01
eGFR, mL/min/1.73m²	97.39 (82.78, 112.03)	92.50 (75.00, 110.50)	101.73 (98.70, 103.00)	<0.01
CysC, mg/L	0.81 (0.69, 0.92)	0.84 (0.70, 1.01)	0.86 (0.75, 0.86)	<0.01

Significant differences in demographic characteristics were observed among the three groups (**Table 1**). Patients in the AS-alone group were younger and predominantly male, whereas a higher proportion of female patients and older age were observed in the autoimmune disease group and the AS comorbidity group (all P < 0.01).

Systemic inflammatory indices differed significantly across groups (**Table 1**). Compared with the autoimmune disease–alone group, patients with AS alone exhibited higher levels of ESR, SII, SIRI, NLR, and PLR. Patients with AS and autoimmune comorbidities demonstrated distinct inflammatory profiles, with several indices differing from both the AS-alone and autoimmune disease–alone groups.

Hematological parameters also showed significant intergroup differences (**Table 2**). WBC, RBC, Hb, HCT, PLT, and related indices differed significantly among the three groups (most P < 0.05). In addition, differential leukocyte counts, including NE, LY, and MO, exhibited distinct distributions across disease groups, with the AS comorbidity group showing patterns that differed from both the AS-alone and autoimmune disease–alone groups.

Liver function parameters differed significantly among the three groups (**Table 3**). TBIL, DBIL, TP, ALB, GLOB, ALB/GLOB, AST, ALT, and TBA showed statistically significant differences across groups (most P < 0.05). Compared with the AS-alone group, patients with AS and autoimmune comorbidities exhibited more pronounced alterations in several liver-related indices.

Renal function and metabolic parameters also varied significantly among the three groups (**Table 4**). BUN, Cr, UA, HCO_3_, eGFR, and CysC differed significantly across groups (all P < 0.01). The AS comorbidity group displayed renal and metabolic profiles that were not entirely consistent with those of the AS-alone or autoimmune disease–alone groups.

To evaluate inter-center consistency, demographic and laboratory variables were compared across the three centers within each disease group, including AS alone, autoimmune diseases alone, and AS with autoimmune comorbidities (**Supplementary Tables 1**-**3**). No significant inter-center differences were observed within the same disease category, indicating minimal center-related heterogeneity. Accordingly, data from the three centers were pooled for subsequent analyses.

### 3.2 Machine learning analysis for discriminating AS alone from AS with autoimmune comorbidities

**Figure 2:**
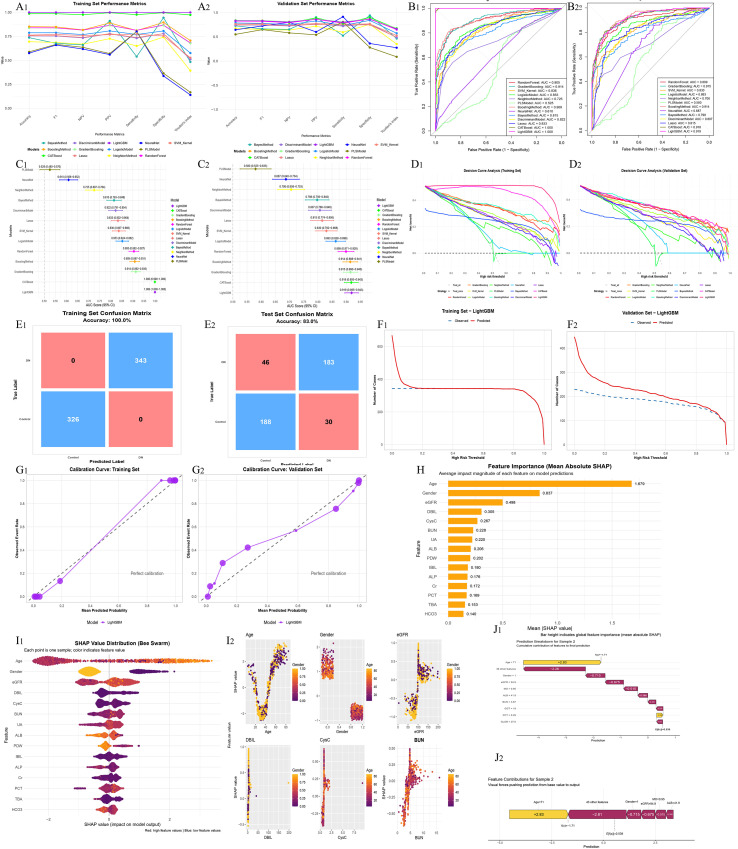
Machine learning–based discrimination between ankylosing spondylitis (AS) alone and AS with autoimmune comorbidities. **(A_1_, A_2_)** Model performance comparison based on accuracy, sensitivity, specificity, F1 score, and AUC. **(B_1_, B_2_)** ROC curves in the training and validation datasets. **(C_1_, C_2_)** Forest plots of AUC values with 95% confidence intervals. **(D_1_, D_2_)** Decision curve analysis (DCA) evaluating clinical net benefit. **(E_1_, E_2_)** Confusion matrices in the training and validation datasets. **(F_1_, F_2_)** Clinical impact curves. **(G_1_, G_2_)** Calibration curves. **(H)** Global SHAP feature importance summary. **(I_1_, I_2_)** SHAP dependence plots for key features. **(J_1_, J_2_)** Individual-level SHAP explanations for a representative patient.

Based on the merged multicenter dataset, multiple machine-learning–based binary classification models were constructed to discriminate patients with AS alone from those with AS combined with autoimmune diseases. Model performance, potential clinical utility, and interpretability were systematically evaluated (**Figure 2**).

Comparisons of overall model performance demonstrated substantial differences among algorithms in terms of accuracy, sensitivity, specificity, F1 score, and AUC (**Figures 2A1, A2**). Overall, ensemble learning models—including LightGBM, random forest, gradient boosting, and CatBoost—outperformed traditional linear models and several single classifiers in both the training and validation datasets. Among these, LightGBM consistently exhibited superior and more stable performance across multiple evaluation metrics.

ROC curve analyses are shown in **Figures 2B1, B2**. LightGBM achieved ROC curves that were consistently above those of other models in both the training and validation sets, with AUC values ranking in the top tier, indicating strong discriminative ability. AUC forest plots further confirmed that LightGBM demonstrated favorable AUC values with relatively narrow confidence intervals, suggesting robust and stable model performance (**Figures 2C1, C2**).

To evaluate potential clinical utility, DCA was performed for all models (**Figures 2D1, D2**). Across a wide range of threshold probabilities, LightGBM provided greater net benefit than other models, supporting its potential usefulness in distinguishing AS alone from AS with autoimmune comorbidities.

Confusion matrices are presented in **Figures 2E1, E2**. LightGBM showed excellent fitting performance in the training set and maintained good classification performance in the test set, indicating acceptable generalizability. Clinical impact curves (**Figures 2F1, F2**) further demonstrated good agreement between the number of patients predicted to be at high risk and the observed outcomes across different threshold probabilities.

Calibration curves for the training and validation datasets are shown in **Figures 2G1, G2**. The curves were generally close to the ideal reference line, indicating good calibration of predicted probabilities generated by the LightGBM model.

After selecting LightGBM as the optimal model, SHAP were applied to interpret model predictions (**Figures 2H–J**). Global feature importance analysis revealed that age, Gender, eGFR, DBIL, CysC, BUN, UA, and ALB were the primary contributors to discriminating AS alone from AS with autoimmune comorbidities (**Figure 2H**).

Integration of SHAP summary plots with the statistical comparisons in **Tables 1**-**4** demonstrated good consistency between feature expression patterns and model-predicted directions. Specifically, older age and female sex were associated with higher SHAP values and tended to drive predictions toward AS with autoimmune comorbidities, with female patients with AS more frequently identified by the model as having additional autoimmune diseases. Regarding renal function–related variables, decreased eGFR and increased levels of CysC, BUN, and UA showed positive contributions to predicting AS with autoimmune comorbidities, whereas patients with AS alone generally exhibited relatively preserved renal function. In addition, elevated DBIL and reduced ALB levels also contributed positively to the prediction of AS with autoimmune comorbidities.

SHAP dependence plots further indicated nonlinear relationships between several key variables and model output, suggesting that the model captured complex feature patterns that are not readily reflected by conventional statistical comparisons (**Figure 2I**). Moreover, individual-level SHAP explanations for a representative patient are illustrated using waterfall and force plots (**Figures 2J1, J2**) to demonstrate the cumulative contribution of individual features to the final prediction. Individual SHAP explanations for additional patients are provided in **Supplementary Figure 2**.

### 3.3 Machine learning analysis for discriminating autoimmune diseases alone from AS with autoimmune comorbidities

**Figure 3:**
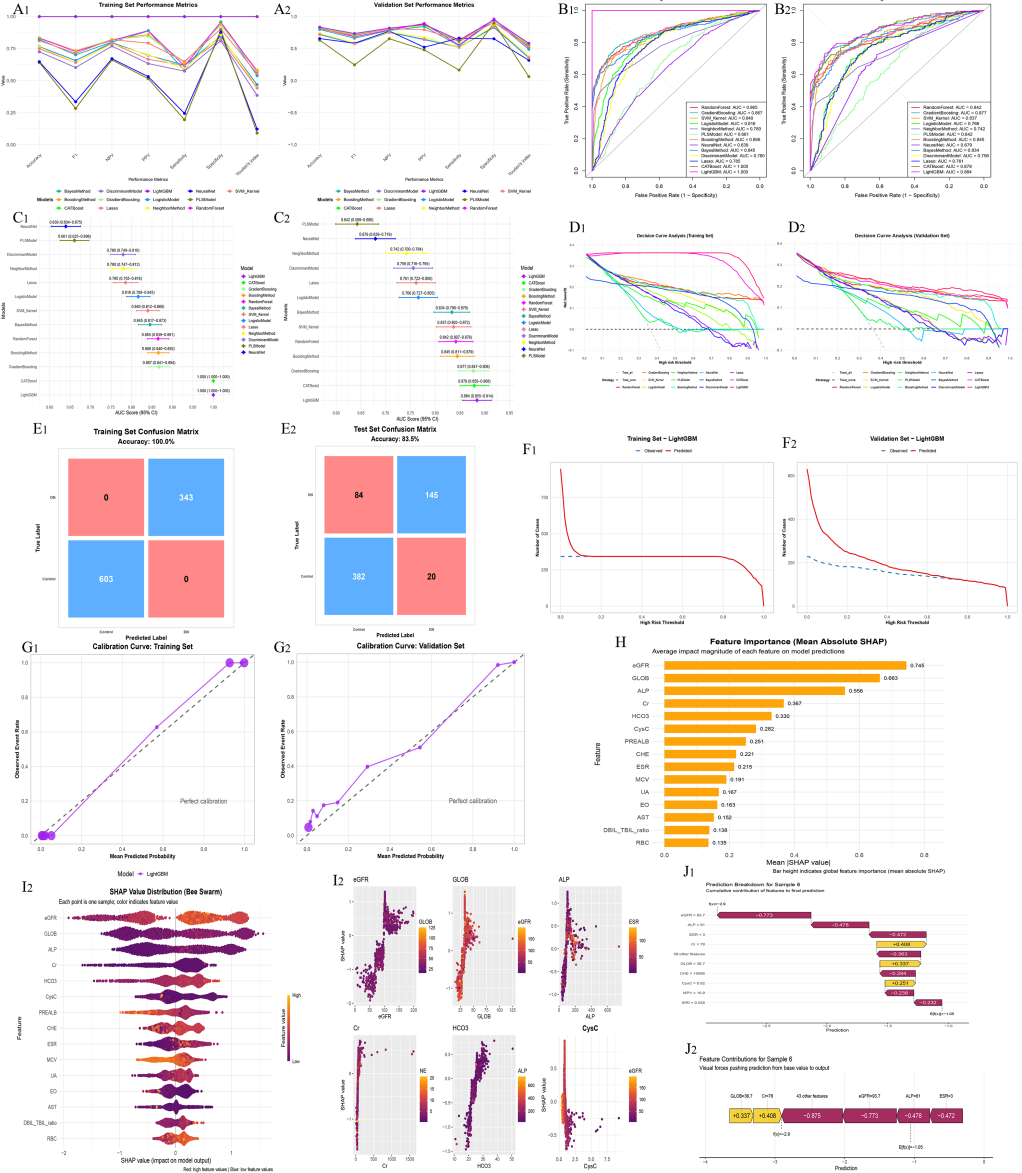
Machine learning–based discrimination between autoimmune diseases alone and ankylosing spondylitis with autoimmune comorbidities. **(A_1_, A_2_)** Model performance comparison based on multiple evaluation metrics. **(B_1_, B_2_)** ROC curves in the training and validation datasets. **(C_1_, C_2_)** Forest plots of AUC values. **(D_1_, D_2_)** Decision curve analysis (DCA). **(E_1_, E_2_)** Confusion matrices. **(F_1_, F_2_)** Clinical impact curves. **(G_1_, G_2_)** Calibration curves. **(H)** Global SHAP feature importance summary. **(I_1_, I_2_)** SHAP dependence plots of key variables. **(J_1_, J_2_)** Individual-level SHAP explanation for a representative patient.

Based on the merged multicenter dataset, multiple machine learning–based binary classification models were further developed to discriminate patients with autoimmune diseases alone from those with AS combined with autoimmune diseases. Model discriminative performance, potential clinical utility, and interpretability were systematically evaluated (**Figure 3**).

Comparisons of overall model performance demonstrated marked differences among algorithms in accuracy, sensitivity, specificity, F1 score, and AUC (**Figures 3A1, A2**). Overall, ensemble learning models outperformed traditional linear models in both the training and validation datasets, among which LightGBM exhibited relatively superior and stable predictive performance across multiple evaluation metrics.

ROC curve analyses are presented in **Figures 3B1, B2**. LightGBM achieved ROC curves that were consistently above those of other models in both the training and validation sets, with relatively high AUC values, indicating strong discriminative ability for distinguishing autoimmune diseases alone from AS with autoimmune comorbidities. AUC forest plots further showed that LightGBM demonstrated favorable AUC values with relatively narrow confidence intervals, suggesting robust model performance (**Figures 3C1, C2**).

To assess potential clinical applicability, decision curve analysis (DCA) was performed for all models (**Figures 3D1, D2**). Across a broad range of threshold probabilities, LightGBM yielded greater net benefit than other models, supporting its potential utility in identifying patients with AS combined with autoimmune diseases.

Confusion matrices are shown in **Figures 3E1, E2**. LightGBM displayed strong fitting performance in the training set and maintained satisfactory classification performance in the test set, indicating acceptable generalizability. Clinical impact curves (**Figures 3F1, F2**) further demonstrated good agreement between the number of patients predicted to be at high risk and the observed outcomes across different risk thresholds.

Calibration curves for the training and validation datasets are presented in **Figures 3G1, G2**. The curves were generally close to the ideal reference line, indicating good calibration of predicted probabilities generated by the LightGBM model.

After selecting LightGBM as the optimal model, SHapley Additive exPlanations (SHAP) were applied to interpret model predictions (**Figures 3H–J**). Global feature importance analysis revealed that eGFR, GLOB, ALP, Cr, HCO_3_^-^, CysC, PREALB, CHE, ESR, and UA were among the most influential variables for discriminating autoimmune diseases alone from AS with autoimmune comorbidities (**Figure 3H**).

Integration of SHAP summary plots with the statistical comparisons in **Tables 1**, **2**, **3**, **4** demonstrated good consistency between feature distributions and model-predicted directions. Specifically, compared with patients with autoimmune diseases alone, those with AS and autoimmune comorbidities exhibited more pronounced abnormalities in renal function–related parameters, including decreased eGFR and increased levels of Cr, CysC, and UA, which showed positive contributions to predicting AS with autoimmune comorbidities in the SHAP analysis (**Table 4**). In addition, protein metabolism and liver function–related variables, such as GLOB, PREALB, CHE, and ALP, also showed substantial contributions in SHAP analysis, consistent with the intergroup differences observed in **Table 3**.

Regarding inflammatory markers, ESR contributed to model discrimination and was generally higher in patients with AS and autoimmune comorbidities, consistent with the statistical differences shown in **Table 1**. SHAP dependence plots further indicated nonlinear relationships between several key variables and model output, suggesting that the model captured complex feature patterns not fully reflected by conventional statistical comparisons (**Figure 3I**). Moreover, individual-level SHAP explanations for a representative patient are illustrated using waterfall and force plots (**Figures 3J1, 2**) to demonstrate the cumulative contribution of individual features to the final prediction. Individual SHAP explanations for additional patients are provided in **Supplementary Figure**.

## 4 Discussion

In this multicenter study, we characterized differences in demographic and laboratory features among AS alone, autoimmune diseases alone, and AS with autoimmune comorbidities (**Tables 1**-**4**). We further built machine-learning models for two classification tasks—AS alone vs. AS with autoimmune comorbidities and autoimmune diseases alone vs. AS with autoimmune comorbidities—and found that LightGBM yielded the most robust performance across AUC, calibration, and decision-analytic metrics (**Figures 1**, **2**). SHAP analyses highlighted consistent key contributors, with feature directions aligning with the intergroup differences observed in **Tables 1**-**4**. These results suggest that routinely collected clinical variables may enable earlier recognition and stratified management of autoimmune comorbidity in AS.

Baseline comparisons revealed that patients with AS combined with other autoimmune diseases exhibited a distinct clinical phenotype compared with patients with AS alone or autoimmune diseases alone. From a demographic perspective, patients in the AS comorbidity group were generally older and more frequently female than those in the AS-alone group, reflecting differences in population distribution across autoimmune disease spectra and suggesting that AS-associated immune comorbidity may preferentially occur in individuals with more complex immune backgrounds (**Table 1**) (17). These demographic characteristics provide clinically relevant clues for risk stratification and early screening of autoimmune comorbidities in AS.

With respect to inflammatory status, systemic inflammatory markers—including ESR and composite indices such as SII, SIRI, NLR, and PLR—demonstrated distinct distribution patterns across the three groups (**Table 1**). While patients with AS alone showed prominent inflammatory features consistent with axial inflammatory disease, the AS comorbidity group displayed an inflammatory profile that was intermediate between, or distinct from, those observed in AS alone and autoimmune diseases alone. This pattern suggests that immune activity in AS with autoimmune comorbidities is not a simple superposition of individual disease processes but rather reflects a more complex, multisystem inflammatory state (18, 19).

Differences in hematological parameters and immune cell composition further supported this observation (**Table 2**). Total leukocyte counts and differential cell distributions, including neutrophils, lymphocytes, and monocytes, varied significantly among disease groups, indicating distinct immune cell proportion patterns (20). Notably, patients with AS and autoimmune comorbidities exhibited hematological features that differed from both comparator groups, consistent with a more heterogeneous and sustained immune-inflammatory milieu (21).

In addition, liver- and kidney-related functional and metabolic parameters showed pronounced intergroup differences (**Tables 3**, **4**). Patients with AS and autoimmune comorbidities demonstrated more systemic alterations across multiple indices of hepatic function, protein metabolism, and renal function. These changes may reflect the cumulative burden of chronic inflammation, the coexistence of multiple immune-mediated conditions, and potential effects of long-term pharmacological treatment or organ involvement (22, 23). Collectively, these findings indicate that AS with autoimmune comorbidities represents a distinct and complex clinical phenotype rather than a simple coexistence of individual diseases, thereby providing a strong clinical rationale for the application of integrative machine learning approaches to facilitate accurate discrimination and risk assessment (24).

In the classification model distinguishing AS alone from AS with autoimmune comorbidities, SHAP analysis demonstrated that age, gender, renal function–related parameters (eGFR, CysC, BUN, and UA), as well as protein metabolism and hepatobiliary indices (ALB and DBIL), were the most influential features contributing to model predictions (**Figure 2**). Notably, the direction and magnitude of these SHAP contributions were highly consistent with the between-group differences observed in **Tables 1**-**4**, thereby validating the findings of conventional statistical analyses at the model level.

With respect to demographic characteristics, SHAP results indicated that older age and female sex were more strongly associated with the coexistence of AS and other autoimmune diseases. This observation is consistent with previous epidemiological reports showing a higher prevalence (25) and greater clinical heterogeneity of autoimmune diseases among females, suggesting that demographic differences may reflect distinct immune backgrounds predisposing to comorbidity (26).

Among laboratory parameters, renal function–related indices ranked prominently in the SHAP analysis. Reduced eGFR together with elevated CysC, BUN, and UA collectively pointed to more pronounced systemic involvement in patients with AS and autoimmune comorbidities. These alterations may be attributable to the cumulative effects of chronic inflammation, immune-mediated microvascular or interstitial injury, and long-term treatment exposure (27, 28). Collectively, these findings indicate that renal function markers provide important discriminative value in differentiating isolated axial inflammatory disease from conditions characterized by multisystem immune involvement (29).

In addition, decreased ALB levels and increased DBIL showed stable contributions in the SHAP analysis, reflecting differences in the inflammation–nutrition status and hepatobiliary metabolic axis in AS with autoimmune comorbidities patients. As a negative acute-phase reactant, reduced ALB commonly indicates sustained inflammatory burden or increased catabolic demand, whereas abnormalities in bilirubin metabolism may be associated with immune-related hepatobiliary involvement or inflammation-driven metabolic remodeling (30, 31).

In comparisons between patients with autoimmune diseases alone and those with AS combined with autoimmune diseases (**Figure 3**), the set of key features identified by SHAP differed markedly from those observed in the analysis of AS alone versus AS with autoimmune comorbidities. This divergence suggests that, in patients already characterized by systemic immune activation, the additional presence of AS is less reflected by further increases in inflammatory intensity and is instead manifested through alterations in metabolic regulation, protein synthetic function, and bone–hepatobiliary–related pathways (32, 33).

Specifically, protein metabolism–related variables, including GLOB, PREALB, and CHE, ranked prominently in the SHAP analysis, indicating more pronounced global differences in immunoglobulin composition, nutritional status, and hepatic synthetic capacity in patients with AS and autoimmune comorbidities (34). Previous studies have shown that sustained inflammation and chronic immune activation in immune-mediated diseases can substantially affect hepatic protein metabolism and synthesis, thereby giving rise to metabolic phenotypes distinct from those observed in isolated autoimmune conditions (35).

In addition, ALP demonstrated a relatively high discriminative contribution in this analysis, suggesting a potential involvement of bone metabolism or biliary-related pathways in AS with autoimmune comorbidities (36). Given that AS is characterized by pathological bone formation and abnormal bone remodeling, the coexistence of AS with other autoimmune diseases may impose more complex inflammatory and immune regulatory influences on the bone–hepatobiliary metabolic axis, resulting in distinctive laboratory signatures (37).

Meanwhile, the prominent contributions of acid–base and metabolic parameters (such as HCO_3_) together with ESR further indicate broader alterations in systemic metabolic regulation and inflammatory activity in patients with AS and autoimmune comorbidities (38). Taken together, these findings suggest that the coexistence of AS with other autoimmune diseases is not merely associated with a quantitative increase in inflammatory burden but rather represents a distinct systemic inflammatory–metabolic phenotype. This phenotypic divergence also provides a plausible explanation for why machine learning models identify different sets of key features across distinct classification tasks.

From a methodological perspective, this study integrated LightGBM with explainable and clinically oriented evaluation frameworks to enhance the robustness and interpretability of the findings. LightGBM effectively captured nonlinear relationships and feature interactions in high-dimensional, heterogeneous clinical data, yielding stable performance across both classification tasks. Model validity was further supported by comprehensive evaluation using ROC/AUC, confusion matrices, calibration curves, decision curve analysis, and clinical impact curves, thereby assessing discrimination, calibration, and potential clinical utility in a unified manner. The application of SHAP enabled both global and individual-level interpretation of model predictions, with representative cases shown in the main figures and additional examples provided in the **Supplementary Materials**. Moreover, the consistency of results across three independent centers (**Supplementary Tables 1**-**3**) reduced center-related bias and supported the generalizability of the proposed models.

In clinical practice, the explainable machine learning models developed in this study, based on routinely available clinical and laboratory variables, may serve as an auxiliary tool to identify AS patients at increased risk of autoimmune comorbidities, thereby prompting targeted symptom assessment and appropriate immunological or imaging evaluations. Conversely, the identified feature patterns may also help recognize patients with autoimmune diseases alone who are at potential risk of concomitant AS, supporting earlier assessment of axial involvement and sacroiliac joint abnormalities. Since all variables used are derived from standard clinical tests, this approach offers good scalability and cost-effectiveness. By leveraging multicenter real-world data, our study provides a novel and practical strategy for the early identification of autoimmune comorbidity in AS.

In this study, patients were divided into three groups: AS alone, autoimmune diseases alone, and AS with autoimmune comorbidities. The rationale behind this grouping was to better assess the clinical differences across these distinct disease states. The clinical rationale for these three-group comparisons lies in the following: Firstly, patients with AS and other autoimmune diseases tend to exhibit unique combined characteristics in their clinical presentation and immune markers, which is distinct from the separate manifestations of AS and autoimmune diseases. Comparing these groups helps reveal the distinct features and clinical signals associated with AS comorbid with autoimmune diseases. Secondly, through this comparison, we aim to assist clinicians in identifying whether an AS patient may have additional autoimmune diseases simply by using basic test data, without the need for expensive or complex diagnostic tools. This approach holds significant practical value for clinical practice. Moreover, the grouping allows us to better understand the clinical characteristics of AS with autoimmune comorbidities, providing further evidence for accurate diagnosis and personalized treatment.

The model developed in this study, based on routinely available clinical and laboratory variables, can be integrated into existing clinical workflows to provide real-time alerts for clinicians. By using simple, accessible test data, the model could help identify AS patients at risk of autoimmune comorbidities early in routine practice. This could guide clinicians to conduct targeted follow-up evaluations, such as immunological assessments or imaging, without requiring additional complex or expensive tests. The model’s practical integration into clinical decision support systems could ultimately enhance early diagnosis and improve personalized treatment strategies for AS patients with immune comorbidities.

Several limitations of this study should be acknowledged. First, the retrospective design based on multicenter real-world data may introduce unavoidable selection bias. Second, patients with missing values were excluded, which could potentially affect the representativeness of the study population; future studies may benefit from advanced strategies such as multiple imputation or explicit modeling of missing-data mechanisms. Third, the spectrum of autoimmune diseases included was heterogeneous, and disease-specific or organ-based subgroup analyses may further refine model performance and clinical interpretability.

In addition, several potentially informative variables, including HLA-B27 status, ANA (anti-nuclear antibody), RF (rheumatoid factor), anti-CCP (anti-cyclic citrullinated peptide antibody), detailed imaging assessments (such as X-ray and MRI), medication exposure (particularly biologic therapies), and standardized disease activity scores (such as BASDAI, DAS28), were not available in the current study. These biomarkers and clinical parameters are critical for more accurate disease classification and prognostic evaluation, and their absence may limit the depth of our analysis. Due to the broad scope of autoimmune diseases included in the study, collecting these specialized diagnostic markers for every disease would have significantly reduced the sample size and introduced potential bias. Therefore, future studies should consider incorporating these key variables to provide more detailed insights into the comorbidities associated with AS.

It is also important to note that while SHAP analysis provides valuable *post hoc* associative explanations of model predictions, it cannot infer causality. The interpretations derived from SHAP are based on the associations present in the data, and may be influenced by issues such as collinearity among features and unmeasured confounding factors in the original dataset. These factors should be considered when interpreting the model’s findings. Prospective validation in independent external cohorts, as well as the development of user-friendly clinical tools, such as online risk calculators, will be important steps toward broader clinical implementation.

## 5 Conclusion

In this multicenter real-world study, we systematically characterized clinical differences among patients with isolated ankylosing spondylitis, autoimmune diseases alone, and ankylosing spondylitis with autoimmune comorbidities, highlighting a distinct composite phenotype associated with immune comorbidity in AS. Across two clinically relevant classification tasks, LightGBM demonstrated robust discriminative performance, stability, and potential clinical utility. Integration of SHAP-based interpretability further identified both shared and task-specific key features contributing to model predictions. Collectively, these findings support the value of explainable machine learning approaches for early identification and risk stratification of autoimmune comorbidity in AS, with promising implications for clinical translation.

## Data Availability

The raw data supporting the conclusions of this article will be made available by the authors, without undue reservation.

## Supplementary material

Supplementary Table 1 Inter-center comparison of demographic and laboratory variables among patients with ankylosing spondylitis alone across the three centers.

Supplementary Table 2 Inter-center comparison of demographic and laboratory variables among patients with autoimmune diseases alone across the three centers.

Supplementary Table 3 Inter-center comparison of demographic and laboratory variables among patients with ankylosing spondylitis combined with autoimmune diseases across the three centers.
