# Supplementary figures and images for: A multicenter explainable machine learning analysis of autoimmune disease comorbidity in ankylosing spondylitis
Source: Front Immunol. 2026 Feb 26;17:1775877. doi: 10.3389/fimmu.2026.1775877 (PMC12979442; doi:10.3389/fimmu.2026.1775877)

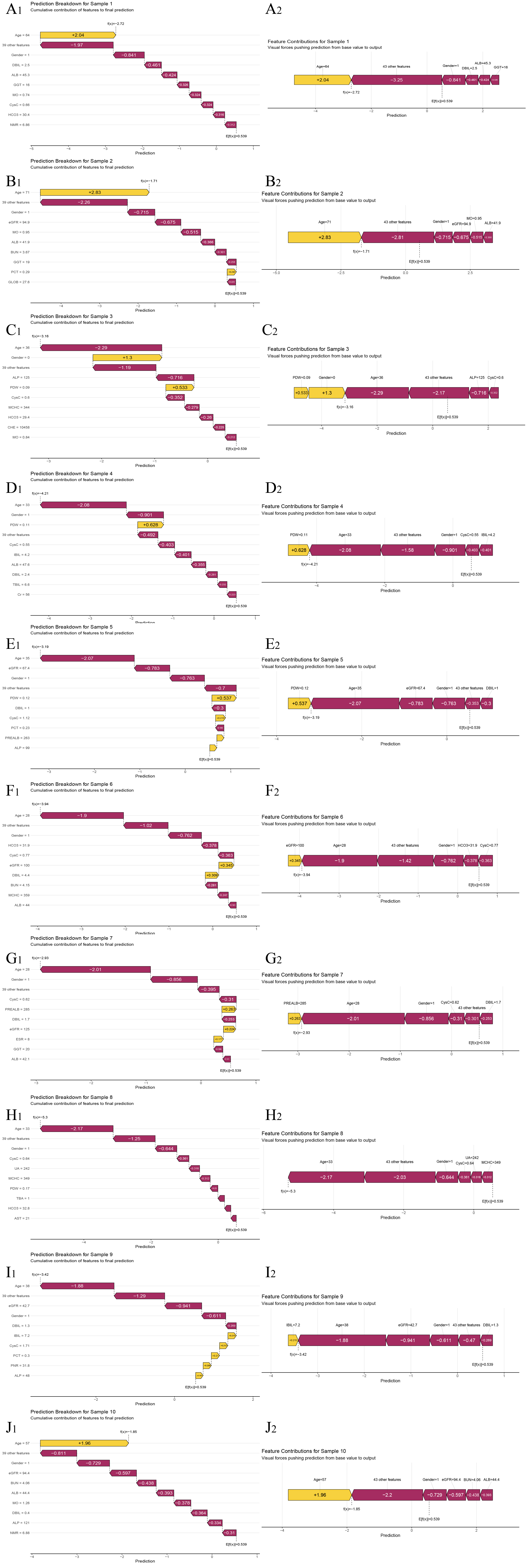

Supplement: Supplementary file 4 [file Image1.jpeg]

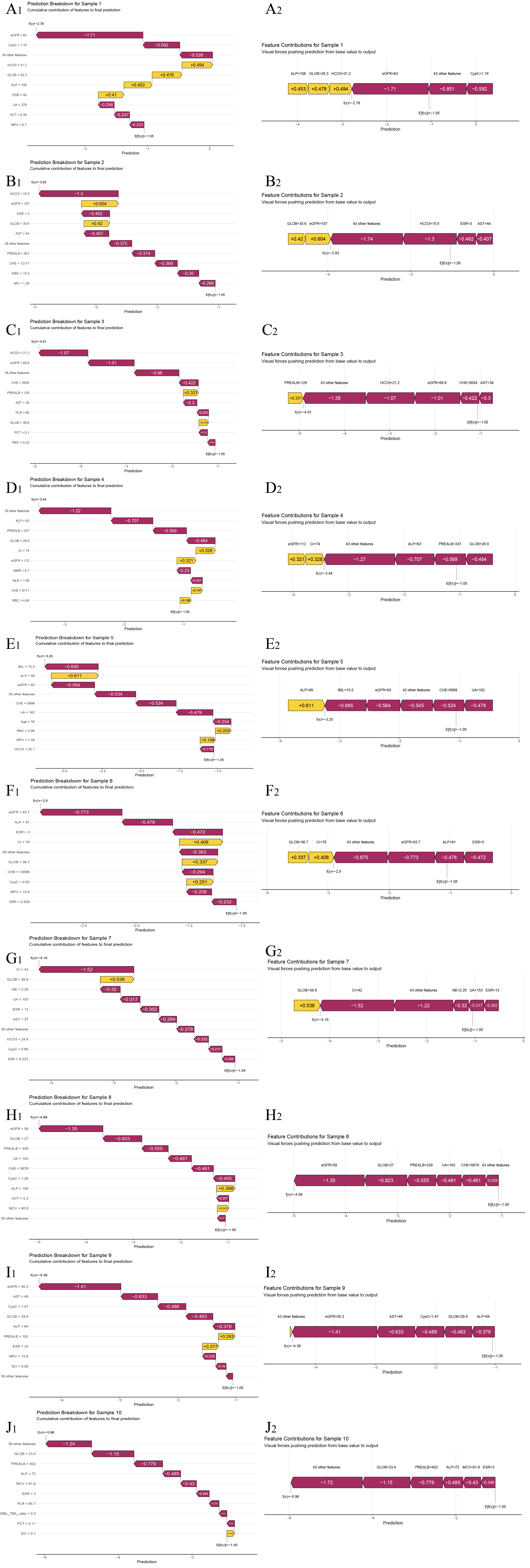

Supplement: Supplementary file 5 [file Image2.jpeg]
